# Surprisingly inflexible: Statistically learned suppression of distractors generalizes across contexts

**DOI:** 10.3758/s13414-021-02387-x

**Published:** 2021-12-03

**Authors:** Jasper de Waard, Louisa Bogaerts, Dirk van Moorselaar, Jan Theeuwes

**Affiliations:** 1 Department of Experimental and Applied Psychology, Vrije Universiteit Amsterdam, Van der Boechorststraat 7, 1081 BT Amsterdam, The Netherlands; 2 Institute Brain and Behavior Amsterdam (iBBA), Amsterdam, the Netherlands

**Keywords:** Statistical learning, Visual attention, Distractor suppression, Context

## Abstract

The present study investigates the flexibility of statistically learned distractor suppression between different contexts. Participants performed the additional singleton task searching for a unique shape, while ignoring a uniquely colored distractor. Crucially, we created two contexts within the experiments, and each context was assigned its own high-probability distractor location, so that the location where the distractor was most likely to appear depended on the context. Experiment 1 signified context through the color of the background. In Experiment 2, we aimed to more strongly differentiate between the contexts using an auditory or visual cue to indicate the upcoming context. In Experiment 3, context determined the appropriate response ensuring that participants engaged the context in order to be able to perform the task. Across all experiments, participants learned to suppress both high-probability locations, even if they were not aware of these spatial regularities. However, these suppression effects occurred independent of context, as the pattern of suppression reflected a de-prioritization of both high-probability locations which did not change with the context. We employed Bayesian analyses to statistically quantify the absence of context-dependent suppression effects. We conclude that statistically learned distractor suppression is robust and generalizes across contexts.

## Introduction

Most theories of attention posit that attentional selection takes place through a combination of *top-down* (voluntary, goal-driven) and *bottom-up* (automatic, stimulus-driven) factors (Corbetta & Shulman, 2002; Jonides, 1981; Posner & Petersen, 1990; Theeuwes, 2010). However, a growing body of literature points to attentional effects that can be explained by neither top-down nor bottom-up processes. To account for these effects, *selection history* was introduced, representing attentional biases that have been learned, often implicitly, from past experience (Awh et al., 2012). It is assumed that the three components of attentional selection (top-down, bottom-up, and selection history) are combined in an integrated priority map, where the input with the highest priority is selected in a winner-takes-all fashion (Theeuwes, 2018). Selection history effects are studied in paradigms such as contextual cueing (Chun & Jiang, 1998; Goujon et al., 2015), reward or punishment learning (Anderson et al., 2011; Della Libera & Chelazzi, 2009; Grégoire et al., 2020), and as of recently statistical learning of distractor suppression (e.g., Ferrante et al., 2018; Wang & Theeuwes, 2018b). Of interest to the current study, contextual cueing, reward learning, and punishment learning have been shown to involve context-dependent learning. For example, stimulus features that have been rewarded in a particular context later only capture attention when presented in the same context (e.g., same background scene; Anderson, 2015). By contrast, a study by Britton and Anderson (2020) found that the implicitly learned spatial suppression of distractors was insensitive to context. Given the theoretical importance of this surprising finding, the current study investigated whether learned distractor suppression might become sensitive to context under circumstances where context is more prominent, or whether, alternatively, the modulation of attentional capture by spatial distractor regularities results in generalized suppression across contexts for different types of context manipulations.

Statistical learning concerns the extraction of regularities in space and time from sensory input (see Frost et al., 2019, for a recent review). Statistical learning research has gained a lot of momentum after the seminal discovery that infants can learn the transitional probabilities from one syllable to the next, facilitating word segmentation (Saffran et al., 1996). Since then, the focus has been extended to adults (Frost et al., 2019), and the statistical learning paradigm was ported to the visual domain by replacing syllables with shapes (Fiser & Aslin, 2002; Turk-Browne et al., 2005). Similarly, spatial relations between shapes and distributional regularities regarding the frequencies of shapes are readily picked up even during passive viewing (Fiser & Aslin, 2001, 2002; Growns et al., 2020). Particularly relevant to the present study is the extraction of regularities concerning distracting stimuli. In the context of visual search, learning the likely properties or location of a distractor can help to decrease distraction, thereby facilitating target detection. Adapting the classic additional singleton paradigm (Theeuwes, 1991), Wang and Theeuwes (2018b) introduced a statistical regularity in the location of the uniquely colored distractor, such that it was far more likely to appear in one location (the high-probability location) than any of the seven other (low-probability) locations in the search display. As a result, participants learned to suppress the high-probability location. This was reflected in faster search times when the distractor appeared on the high-probability location and slower search times when the target did (see also Ferrante et al., 2018; van Moorselaar & Slagter, 2019). Crucially, an explicit knowledge test at the end of the experiment indicated that learning had taken place in the absence of awareness (but see Vadillo et al., 2016).

Context plays a major role in many theories of learning and memory (e.g., Godden & Baddeley, 1975), and history-based attentional biases have also been suggested to apply “when the relevant context is encountered” (Awh et al., 2012). There is a vast advantage of context-dependency. Context-*independent* learning can only be short-lived, requiring a constant re-learning of biases for contexts that have already been encountered, whereas context-*dependent* learning allows learned regularities to be stored while new ones are being learned or updated. It should come as no surprise then, that reward learning (Anderson, 2015; Anderson & Kim, 2018), punishment-based learning (Grégoire et al., 2020), and contextual cueing (Brooks et al., 2010; Jiang & Song, 2005) have been shown to be context dependent. In contextual cueing, a *hyper specificity* was reported, with no transfer at all between contexts that only differed in color (Jiang & Song, 2005). Furthermore, the application of different search modes is also context-dependent (Cosman & Vecera, 2013). Lastly, the finding that statistical learning of transitional regularities can be retained for up to 1 year (Arciuli & Simpson, 2012; Kim et al., 2009; Kóbor et al., 2017) is suggestive of context sensitivity; if insensitive to context, those regularities would have long been replaced by more recently learned ones. There are of course many differences between these paradigms and statistically learned suppression. Notably, studies on reward or punishment-based learning and contextual cueing all involved target-based as opposed to distractor-based learning, which might operate on different processes (Di Caro & Della Libera, 2021; Turatto & Pascucci, 2016; Won & Geng, 2020), and findings from other areas within statistical learning do not necessarily translate to distractor suppression. Nevertheless, the overall picture is one where the benefits of context-dependent learning are found across a range of implicit learning paradigms.

While the arguments presented above would lead to a prediction of context-dependent statistical learning of distractor suppression, a recent study by Britton and Anderson (2020) did not find evidence for these effects. Using an adapted version of the paradigm by Wang and Theeuwes (2018b), they reported suppression effects that generalized across contexts. In their study (Experiment 1), the context on each trial was determined by a grayscale background image of a forest or a city (as in the prior study by Anderson, 2015, on reward learning). Crucially, the high-probability distractor location depended on the context, so that the urban background predicted a different distractor location than the forest. The results indicated that learning had taken place: response times (RTs) were faster when the distractor was at a high-probability versus a low-probability location, even though participants had no awareness of the spatial regularities. However, this learning was insensitive to context. Between the two high-probability distractor locations, RTs were the same whether predicted by the context or not.

Given the discrepancy between advantages of context-dependent learning and the context-dependent effects in related paradigms on the one hand, and the context generalization in Britton and Anderson’s (2020) experiment on the other hand, we conducted three experiments to verify the conclusion that statistically learned distractor suppression generalizes across contexts within a task. Similar to Britton and Anderson’s (2020) experiment, in each of our experiments two contexts had their own high-probability distractor location, and these two locations were maximally distant. Experiment 1 conceptually replicated Britton and Anderson’s study, but signified context through the brightness of the background. To increase the subjective difference between the two contexts, in Experiment 2 we employed an auditory versus a visual cue. In Experiment 3, we coupled each context with a different response mapping, so that processing the context was a necessity for performing the task.

## Experiment 1

Experiment 1 was a conceptual replication of Britton and Anderson’s (2020) study (Experiment 1). The background (visible throughout a trial) was light grey in one context, and dark grey in the other context, so that distinguishing between contexts would be effortless and fast. Each context had its own high-probability distractor location, which remained constant throughout the experiment. Following reports of a suppression gradient around the high-probability distractor location (e.g., Wang & Theeuwes, 2018b), the two high-probability locations were kept maximally distant. We used eight rather than six stimuli to increase the salience of the distractors and avoid any potential serial search effects.

**Fig. 1:**
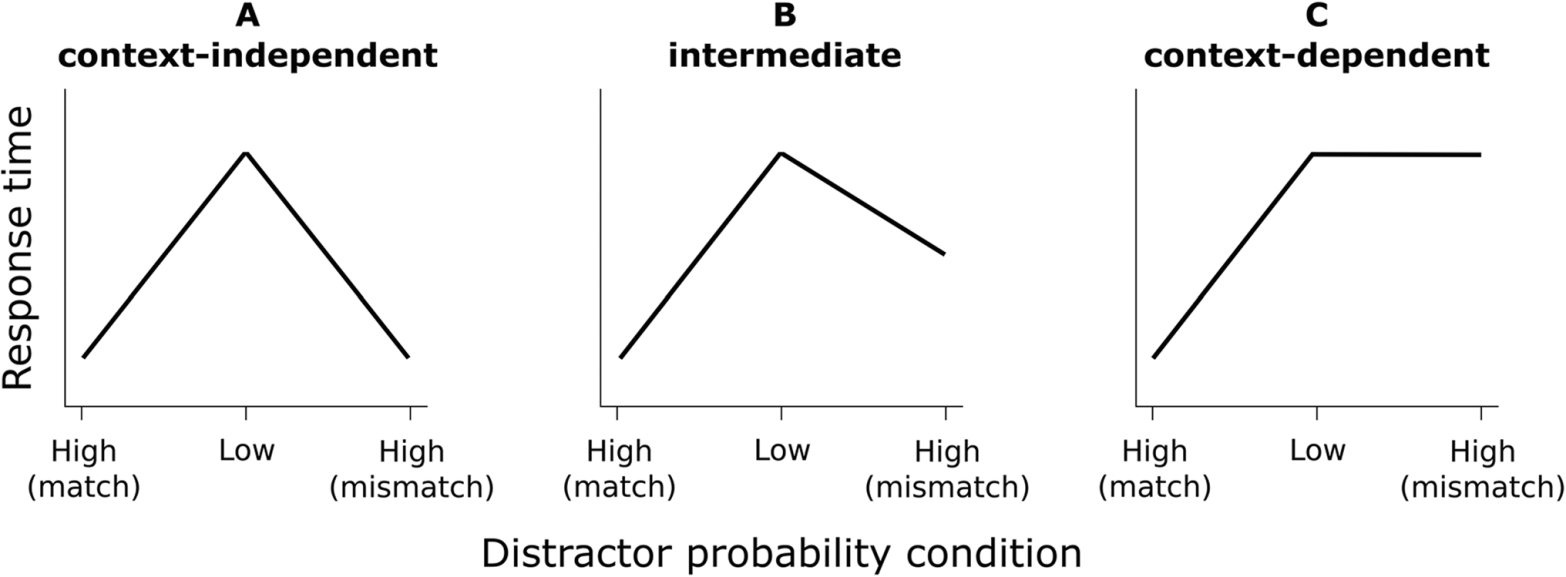
Simplified possible outcomes of Experiment 1. The ordering of the labels on the x-axis is based on distance; the two high-probability locations were always maximally distant. (**A**) Learning is context-independent, so that each high-probability location is suppressed equally. (**B**) Learning is somewhat context-dependent, so that the high-probability location matching the current context is suppressed most. (**C**) Learning is fully context-dependent, so that suppression occurs only for the matching high-probability location

The three distractor location conditions of interest were low-probability (a distractor on any of the less frequent locations), high-probability match (a distractor on the high-probability location of the current context), and high-probability mismatch (a distractor on the high-probability location of the other context). Figure 1 illustrates three possible outcomes. If learning is context-independent, each high-probability location should be suppressed equally, irrespective of whether it matches or mismatches with the current context (A). If learning is fully context-dependent, suppression should occur only for the high-probability match condition and RTs for the high-probability mismatch condition should roughly equal those for low-probability (C). Finally, if learning is partially context-dependent, we predict the fastest RTs for the high-probability match condition, but at the same time faster RTs for the high-probability mismatch condition as compared to the low-probability condition (B).

## Methods

All experiments were approved by the Ethical Review Committee of the Faculty of Behavioral and Movement Sciences of the Vrije Universiteit Amsterdam*.*

### Participants

Sixty-one adults (32 male, 27 female, one non-binary, one unknown, mean age = 30 years, age range: 20–46) participated in an online experiment through Prolific (Palan & Schitter, 2018). They all reported having normal or corrected-to-normal (color) vision, and at minimum an undergraduate degree. Participation took ± 30 min and participants earned £3.75. Following Britton and Anderson (2020), an effect with *d* = 0.6 (taken from Failing et al., 2019) would require a sample size of 31 to get β = 0.90 when α = 0.05. However, since they did not find a significant result, we attempted to detect a smaller effect size (*d* = 0.45), which required a sample size of 54 to get β = 0.90 when α = 0.05. The number of non-discarded participants exceeded this minimal sample size in all experiments.

### Apparatus and stimuli

Because the experiment took place online, some factors (e.g., lighting and seating conditions) could not be controlled. For replication purposes, item sizes and colors are reported in pixels and RGB values (red/green/blue). The experiment was created in OpenSesame (Mathôt et al., 2012) using OSweb, and run using JATOS (Lange et al., 2015).

**Fig. 2:**
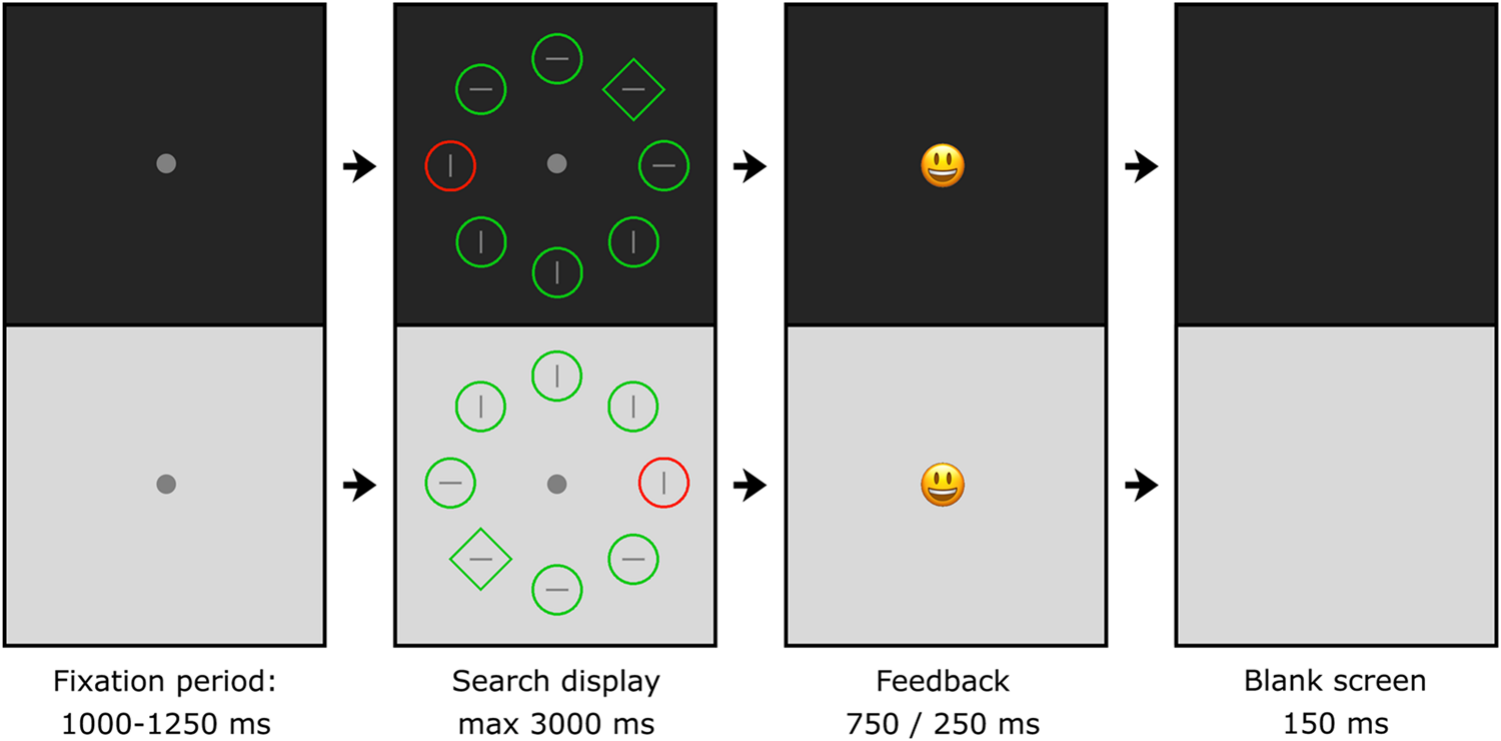
Schematic overview of a trial, with the top presenting one context and the bottom the other context. The background color remained constant throughout a trial. The search display was visible until a keyboard response was provided or the 3000 ms limit was exceeded. Participants indicated the orientation of the line inside the unique shape by pressing the up or left arrow key (timed). A smiley provided feedback immediately after the response was given.

The experimental display for the two contexts is illustrated in Fig. 2. It consisted of eight shapes (one circle and seven diamonds, or vice versa), presented on an imaginary circle with a radius of 224 px. Each shape contained a grey (128/128/128) vertical or horizontal line (49 × 7 px). The circles and diamonds were 108 and 134 px high, respectively, in red (255/0/0) or green (0/200/0). Depending on the context, the background was light (204/204/204) or dark (51/51/51) grey. The fixation dot was grey (153/153/153, radius: 7 px).

### Procedure and design

Figure 2 gives a schematic overview of a trial. The duration of the fixation period was randomly selected between 1,000 and 1,250 ms. The search display was visible until response or until a 3,000-ms limit was exceeded. Participants searched for the unique shape (i.e., a circle among diamonds or vice versa), and indicated the orientation of the line segment inside (horizontal/vertical) by pressing the up or left arrow key as quickly as possible. Subsequently, a smiley provided positive (250 ms) or negative (750 ms) feedback, followed by a blank screen (150 ms). The longer duration of negative feedback ensured that participants who aimed to finish the experiment quickly would benefit from providing correct responses. The background color, distinguishing between contexts, remained constant and at all times visible throughout a trial.

The target was always the uniquely shaped item, while the distractor was the uniquely colored item. Each context occurred equally often. A target was present on each trial, containing a line that was vertical or horizontal at random. A uniquely colored distractor was present on 84% of the trials. The distractor could be present at any of the eight locations. However, within each context, one distractor location occurred more often (67%) than the other locations (4.7% per location). The two high-probability locations (one for each context) were determined randomly for each participant, with the high-probability location of one context always opposite to that of the other context. The target location was determined randomly on each trial. Participants completed 20 practice trials, followed by four blocks of 125 trials each. A break was included after every block, and trial order was randomized within blocks. Awareness of the spatial regularities was assessed after all trials were completed by asking participants whether the distractor appeared more frequently in one location, and secondly to indicate this location in four trials (context A/B × circle/diamond-shaped target) by typing in a location-based number (1–8).

## Results

The data for one participant were discarded because the experiment was not fully completed. A further five participants were discarded because their accuracy scores were below 75%. Incorrect trials (4.2% of trials) and trials on which the RTs were slower than 2,000 ms (4.8% of trials) were excluded from all further analyses. As there was no evidence in support of a speed-accuracy trade-off (neither here, nor in Experiments 2 and 3), we only report RT results.

First, to test if the statistical regularity in distractor location probability (across contexts) is modulating suppression, we performed a repeated-measures ANOVA. Second, to test whether there is evidence for context-dependent statistical learning, we performed a t-test analysis comparing high-probability match and high-probability mismatch trials. In order to uncover the strength of evidence for the null-hypothesis, we performed a Bayesian t-test for the same comparison. Finally, we analyze participants’ awareness of the regularities using Bayes factors.

ANOVAs and t-tests were performed using Jamovi (Sahin & Aybek, 2019). Note that due to a violation of the sphericity assumption, a Greenhouse-Geisser correction was applied to the ANOVA results. Bayesian analyses were performed in JASP (JASP Team, 2020), using the default Cauchy distribution (scale = 0.707) as the prior. Reported Bayes factors reflect the ratio of the likelihood of the null-hypothesis H_0_ relative to the alternative hypothesis H_1_ (i.e., BF_01_).

### Statistical learning: Are search times modulated by distractor probability?

**Fig. 3:**
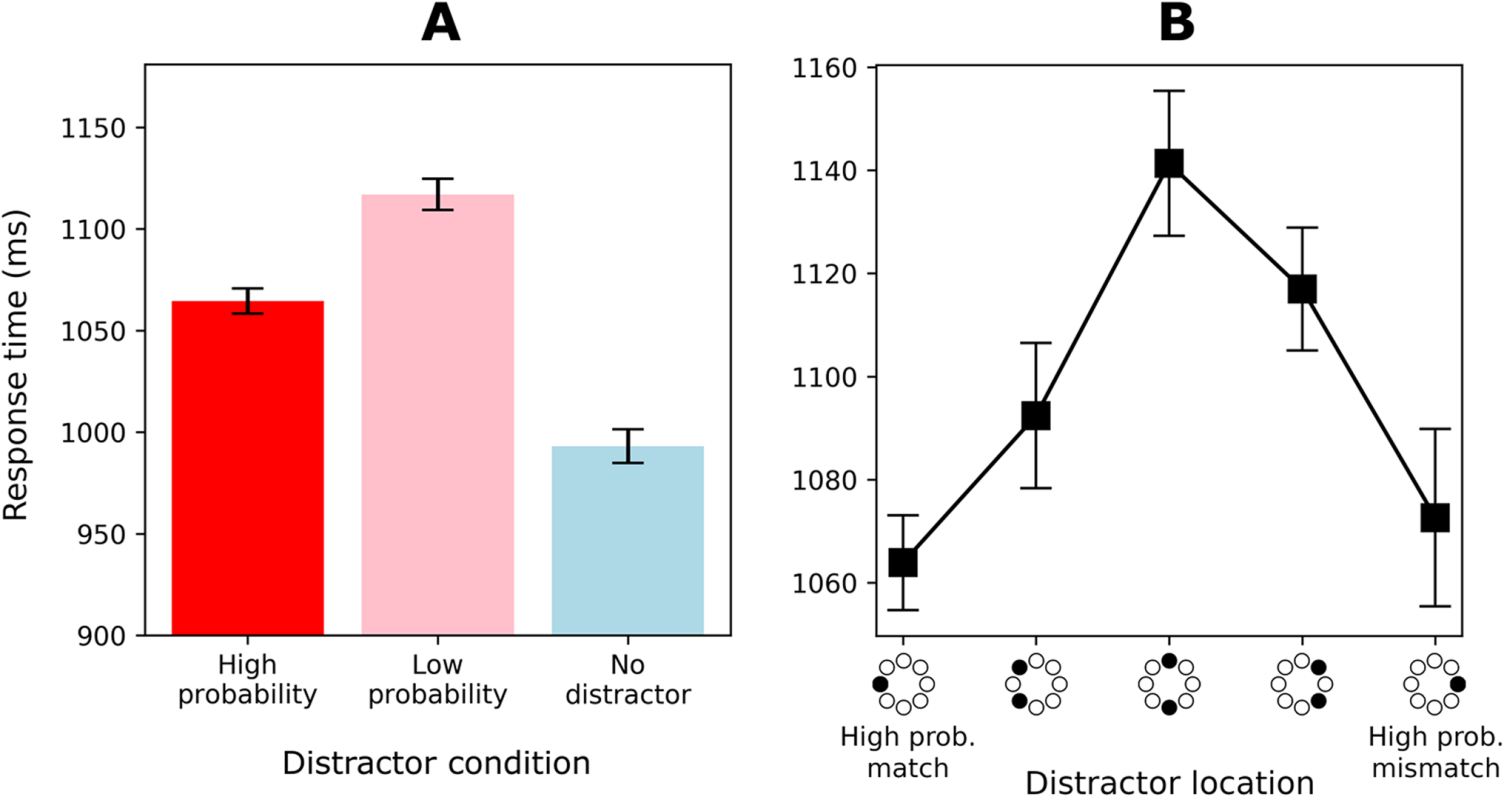
Response time (RT) results of Experiment 1. Error bars indicate 95% within-subject confidence intervals (Cousineau, 2005). (**A**) Mean RTs for high-probability, low-probability, and no-distractor conditions. Note that “high probability” refers to both high-probability locations (irrespective of context), and “low probability” refers to the six remaining locations. (**B**) Mean RTs as a function of distractor location, coded as the distance from the high-probability location of the current context (high-probability match)

Figure 3A shows mean RTs for the high-probability (in either context), low-probability, and no-distractor conditions. A one-way repeated-measures ANOVA with the factor *distractor condition* showed a significant main effect on RTs, *F*(1.81, 97.76) = 177, *p* < .001, partial *η*^*2*^ = 0.767. Planned comparisons showed that the distractor captured attention reliably for both the low-probability, *t*(54) = 16.41, *p* < .001, *d* = 1.51, and high-probability locations, *t*(54) = 11.22, *p* < .001, *d* = 2.21. Furthermore, they reveal a reliable difference between the high- and low-probability locations, *t*(54) = 9.10, *p* < .001, *d* = 1.23, indicating that participants learned the overall regularities in the experiment.

### Is the learned distractor suppression context-dependent?

Figure 3B shows the mean RTs for distractor location in greater detail. To investigate whether the distractor suppression was context-dependent, distractor location is coded as the distance from the high-probability location of the current context (high-probability match). As outlined in Fig. 1, the relevant comparison for investigating context-dependency is between high-probability match and high-probability mismatch trials. A paired t-test reveals that this difference was nonsignificant, *t*(54) = 0.77, *p = .*445, *d* = 0.10, *BF* = 5.13. This *BF* indicates that the observed data are about five times more likely to have occurred under the null hypothesis, providing substantial (Jeffreys, 1998) evidence that the learned distractor suppression was not context-dependent. RTs for the high-probability mismatch location were faster than for the low-probability locations, *t*(54) = 3.94, *p < .*001, *d* = 0.53, indicating that the mismatch location was indeed suppressed.

### Awareness of the regularities

About half of the participants (47%) answered “yes” to the question if the distractor occurred more often at some locations than others. An awareness score was computed for every participant by taking the average distance between the location indicated by the participant and the actual high-probability distractor location on the four awareness trials. The mean awareness score across participants was 2.09 (SD = 0.53). A one-sided Bayesian t-test comparing awareness scores against chance level (2.0) yielded a *BF* of 12.4 in favor of no awareness. Furthermore, we found no difference in awareness scores between participants who answered “yes” versus those who answered “no” on the first question, *t*(53) = 0.95, *p = .*345, *d* = 0.26, *BF* = 2.52. We also repeated all previous RT analyses separately for the “yes” and the “no” group, and found no differences in the pattern of results. Most importantly, there was no difference in RTs between the high-probability match and mismatch conditions in the “yes” group, *t*(25) = 1.21, *p = .*239, *BF* = 2.51, *d* = 0.24, and in the “no” group, *t*(28) = 0.07, *p = .*942, *BF* = 5.05, *d* = 0.01. We conclude that participants had no or very little awareness of the learned regularities.

## Discussion

The results of Experiment 1 indicate that statistical learning of distractor suppression is independent of context. Replicating Wang and Theeuwes (2018a, b) we observed that compared to low-probability locations, RTs were faster when the distractor was at a high-probability location. This indicates that participants learned the overall regularities of the experiment. Crucially, however, there was no context-specific suppression effect; participants responded equally fast when the distractor location matched the current context (high-probability match) or matched the other context (high-probability mismatch). This finding is in line with Britton and Anderson (2020). With regard to the predictions of Fig. 1, the results are consistent with what is displayed in panel A, indicating that the learned suppression of a probable distractor location generalized across contexts.

Analyses on awareness indicate that, at the group level, participants were likely unaware of the regularities (although caution in the interpretation is warranted, see Vadillo et al., 2016). We conclude that the suppression of distractor locations is most likely the consequence of implicit learning.

## Experiment 2

In Experiment 2 we attempted to increase the chances of finding a significant context-dependent suppression effect. Since learned distractor suppression is proactive (Huang et al., 2021), context-specific suppression necessarily relies on an effective instantiation of context through the cue. In the domain of temporal preparation, Los et al. (2021) showed that a between-modalities cue is more effective in producing a temporal context than a within-modalities cue. We reasoned that this might also apply to spatial statistical learning. Therefore, we used a cue that was either a flashed ring or a tone, presented at the start of each trial. To further increase our chances of observing a significant effect, we decreased the smallest effect size that we would be able to detect to d = 0.35 by increasing the amount of non-discarded participants to 95.

## Methods

### Participants

A power analysis revealed that in order to detect an effect of d = 0.35, we needed a sample size of at least 88 to get β = 0.90 when α = 0.05. The number of non-discarded participants in Experiment 2 exceeded this minimal sample size. In total, 114 adults (54 male, 55 female, three non-binary, two unknown, mean age = 28 years, age range: 18–55) participated online through Prolific. Inclusion criteria were identical to Experiment 1. Participation took ± 35 min, with a reward of £4.38.

### Apparatus and stimuli

**Fig. 4:**
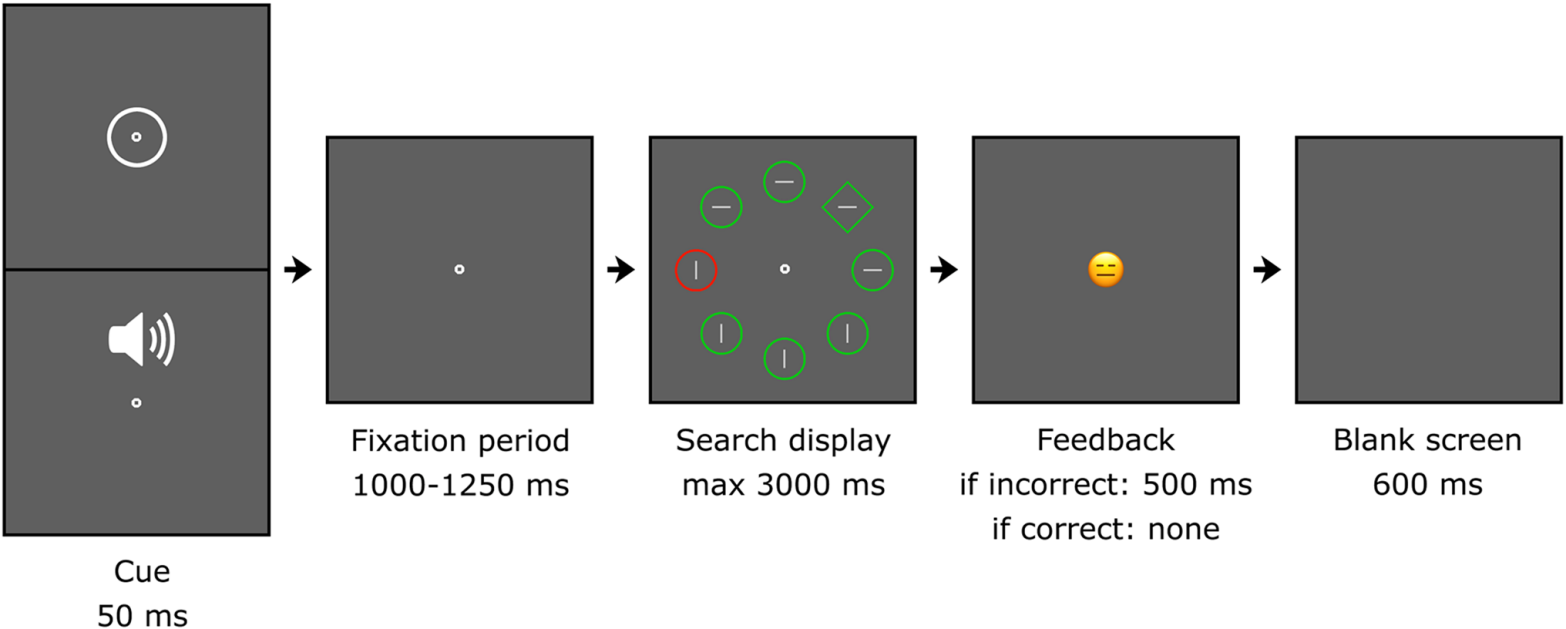
Schematic overview of a trial. The cue was a flashed ring or a tone. The search display was visible until a keyboard response was provided or the 3,000-ms limit was exceeded. Participants indicated the orientation of the line inside the unique shape by pressing the up or left arrow key (timed). Feedback was provided in the form of a frowning smiley after an incorrect response

The experimental apparatus was identical to Experiment 1. The stimuli are illustrated in Fig. 4. The shapes and colors were identical to Experiment 1, with the exceptions that the fixation dot (radius: 7 px) was white, and the background was dark grey (94/94/94). The contextual cue was a white ring (radius: 35 px, thickness: 3 px) or a tone (500 Hz, -10 dB).

### Procedure and design

Figure 4 gives a schematic overview of a trial. The task and stimulus timings were identical to Experiment 1, with the addition of a visual or auditory cue (50 ms) at the start of every trial. To save time, feedback (a frowning smiley, 500 ms) was only provided after an incorrect response. To clearly segregate each trial from the following trial (and thereby each context from the following context), a blank screen of 600 ms was included at the end of each trial.

One context was cued by a flashed ring, the other by a tone. The distribution of the target and distractor locations across trials was identical to Experiment 1. Before the start of the experiment, participants were given the opportunity to adjust their sound to a comfortable level. To ensure this was loud enough and functioning as expected, the tone was subsequently tested in a short task. The tone was presented ten times with random time intervals, and participants responded to each instance by pressing space (timed). To ensure that participants did not turn off their sound during the experiment, an audio check was included at the start of a random trial in every block of 50 trials. Each audio check displayed the text “audio check” in red for 1,500 ms. A sound (identical to the auditory cue) was presented after 500 ms during half of the audio checks, and participants had to indicate whether it was present or absent by pressing the Y or N key, respectively. Participants completed 20 practice trials, followed by four blocks of 125 trials each. A break was included after every block, and trial order was randomized within blocks. Awareness of the spatial regularities was assessed similarly to Experiment 1, after all trials were completed.

## Results

The data for two participants were discarded because they did not complete the experiment. A further four participants were discarded because our built-in sound check indicated that they had turned off their sound, and 13 participants were discarded because their accuracy scores were below 75%. Incorrect trials (5.5% of trials) and trials on which the RTs were slower than 2,000 ms (5.7% of trials) were excluded from all further analyses. The following analyses are identical to those of Experiment 1.

### Statistical learning: Are search times modulated by distractor probability?

**Fig. 5:**
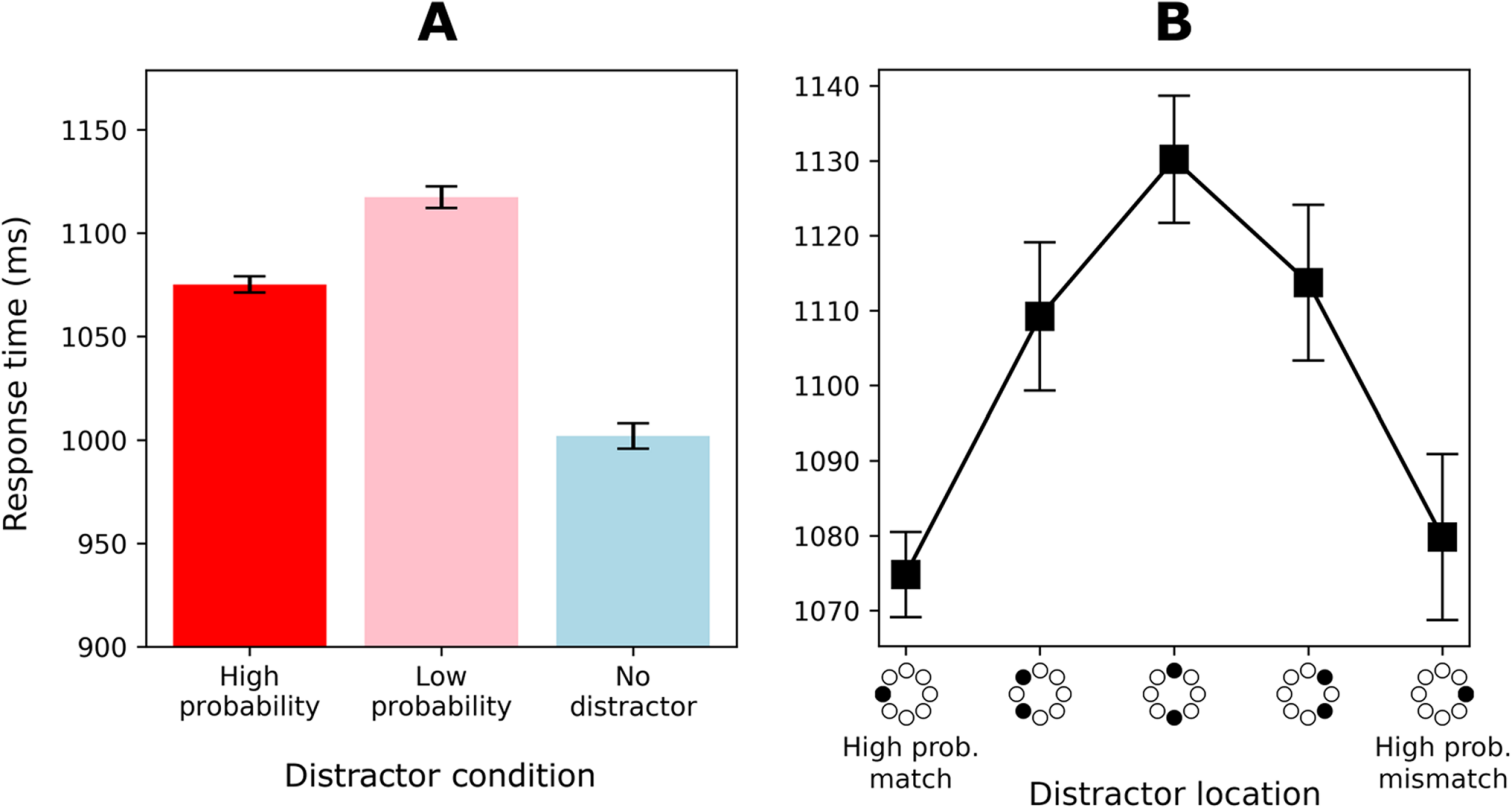
Response time (RT) results for Experiment 3. Error bars indicate 95% within-subject confidence intervals (Cousineau, 2005). (**A**) Mean RTs for high-probability, low-probability, and no-distractor conditions. Note that “high probability” refers to both high-probability locations (irrespective of context), and “low probability” refers to the six remaining locations. (**B**) Mean RTs as a function of distractor location, coded as the distance from the high-probability location of the current context (high-probability match)

Figure 5A shows mean RTs for the high-probability, low-probability, and no-distractor conditions. The factor *distractor condition* showed a main effect, *F*(1.63, 153.1) = 328, *p* < .001, partial *η*^*2*^ = 0.777. Planned comparisons showed that the distractor captured attention reliably for both the low-probability, *t*(94) = 21.2, *p* < .001, *d* = 2.18, and high-probability locations, *t*(94) = 16.1, *p* < .001, *d* = 1.65. Furthermore, they showed a reliable difference between the high- and low-probability locations, *t*(94) = 12.2, *p* < .001, *d* = 1.25, indicating that participants learned the overall regularities in the experiment.

### Is the learned distractor suppression context-dependent?

Figure 5B shows the mean RTs for distractor locations in greater detail. The comparison between high-probability match and high-probability mismatch trials was not significant, *t*(94) = 0.29, *p = .*774, *d* = 0.03, *BF* = 8.46. This again provides substantial (Jeffreys, 1998) evidence that the learned distractor suppression was not context-dependent. RTs for the high-probability mismatch location were faster than for the low-probability locations, *t*(94) = 5.35, *p < .*001, *d* = 0.55, indicating that the mismatch location was indeed suppressed.

### Awareness of the regularities

The results regarding participants’ awareness of the regularities are in line with those of Experiment 1. About half of the participants (46%) indicated that the distractor occurred more often at some locations than others. The mean awareness score across participants was 1.96 (SD = 0.43), yielding a *BF* of 15.7 in favor of no awareness. Furthermore, we found no difference in awareness scores between participants who answered “yes” versus “no” on the first question, *t*(93) = 0.11, *p = .*911, *d* = 0.02, *BF* = 4.61. We also repeated all previous RT analyses separately for the “yes” and the “no” groups, and found no differences in the pattern of results. Most importantly, there was no difference in RTs between the high-probability match and mismatch conditions in the “yes” group, *t*(43) = 0.15, *p = .*882, *BF* = 6.06, *d* = 0.02, and in the “no” group, *t*(50) = 0.28, *p = .*781, *BF* = 6.33, *d* = 0.04. We conclude again that participants had no or very little awareness of the learned regularities.

## Discussion

In line with Experiment 1, Experiment 2 showed no context-specific suppression effects. Again, there was clear evidence that participants learned to suppress the high-probability distractor locations, but this suppression was generalized across contexts. As in Experiment 1, learning likely occurred outside of awareness.

## Experiment 3

We observed no context-dependent suppression effects in Experiments 1 and 2. However, context arguably played a relatively small role in both experiments. In Experiment 1, it was possible to go through the entire experiment without paying any mind to context (the brightness of the background), and in Experiment 2 context (a flashed ring or a tone) was probed only indirectly through the sound checks (ten in total). In Experiment 3, therefore, we intertwined context with the experimental task such that ignoring it would no longer be an option. To achieve this, we made the correct response dependent on the context. In the ZC-context, participants responded to the orientation of the target by pressing the Z or C key, and in the arrow-context they used the (left/right) arrow keys. We returned to the sample size estimation from Experiment 1.

## Methods

### Participants

Sixty adults (27 male, 29 female, four non-binary, mean age = 26.5 years, age range: 20–42) participated in an online experiment through Prolific (Palan & Schitter, 2018). They all reported having normal or corrected-to-normal (color) vision, and at minimum an undergraduate degree. Participation took ± 30 min and participants earned £3.75.

### Apparatus and stimuli

**Fig. 6:**
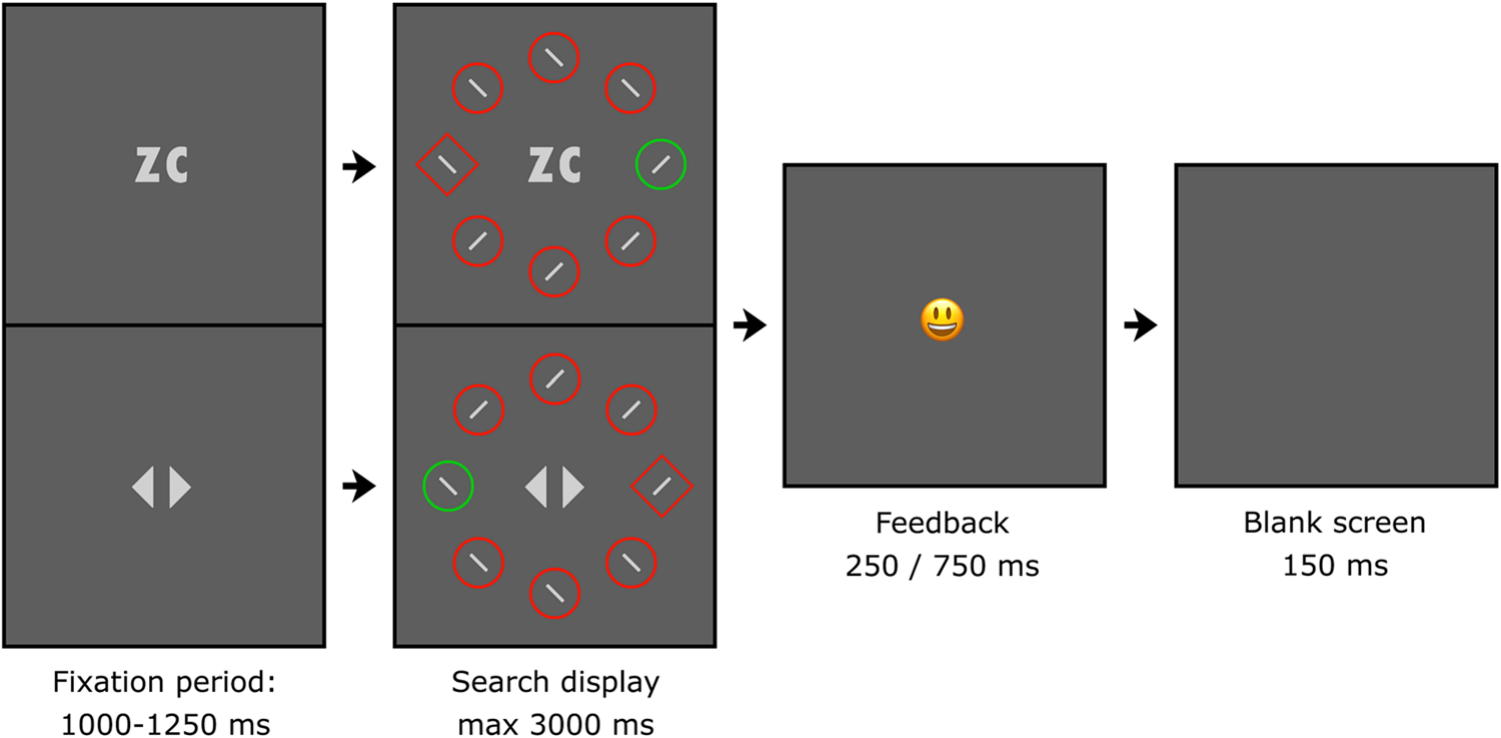
Schematic overview of a trial. The cue was a light grey ZC or two triangular arrows. The search display was visible until a keyboard response was provided or the 3,000-ms limit was exceeded. Participants indicated the orientation of the line inside the unique shape by pressing either the Z or C key, or the left or right arrow key (timed). A smiley provided feedback immediately after the response was given

The experimental apparatus was identical to Experiment 1. The stimuli are illustrated in Fig. 6. The stimuli were identical to Experiment 1, with the exceptions that all grey lines were 45° tilted, the fixation dot was replaced by the grey letters ZC or two triangular arrows (128/128/128, height: 80 px), and the background was dark grey (94/94/94).

### Procedure and design

Figure 6 gives a schematic overview of a trial. The task and stimulus timings were identical to Experiment 1, with the exception that the correct response depended on the context. In the ZC-context, participants responded with a Z to a left-tilted target and a C to a right-tilted target. In the arrow-context, participants responded to the left or right-tilted target by using the left or right arrow key.

The distribution of the target and distractor locations across trials was identical to Experiment 1. Because this task was harder than Experiments 1 and 2, participants first completed 30 practice trials, and were forced to repeat the practice round until they reached at least 75% accuracy. The experimental trials consisted of four blocks of 125 trials each. A break was included after every block, and trial order was randomized within blocks. Awareness of the spatial regularities was assessed similarly to Experiment 1, after all trials were completed.

## Results

The data for three participants were discarded because their accuracy was below 75%. Incorrect trials (10.3% of trials) and trials on which the RTs were slower than 2000 ms (8.8% of trials) were excluded from all further analyses. The following analyses are identical to those of Experiment 1.

### Statistical learning: are search times modulated by distractor probability?

**Fig. 7:**
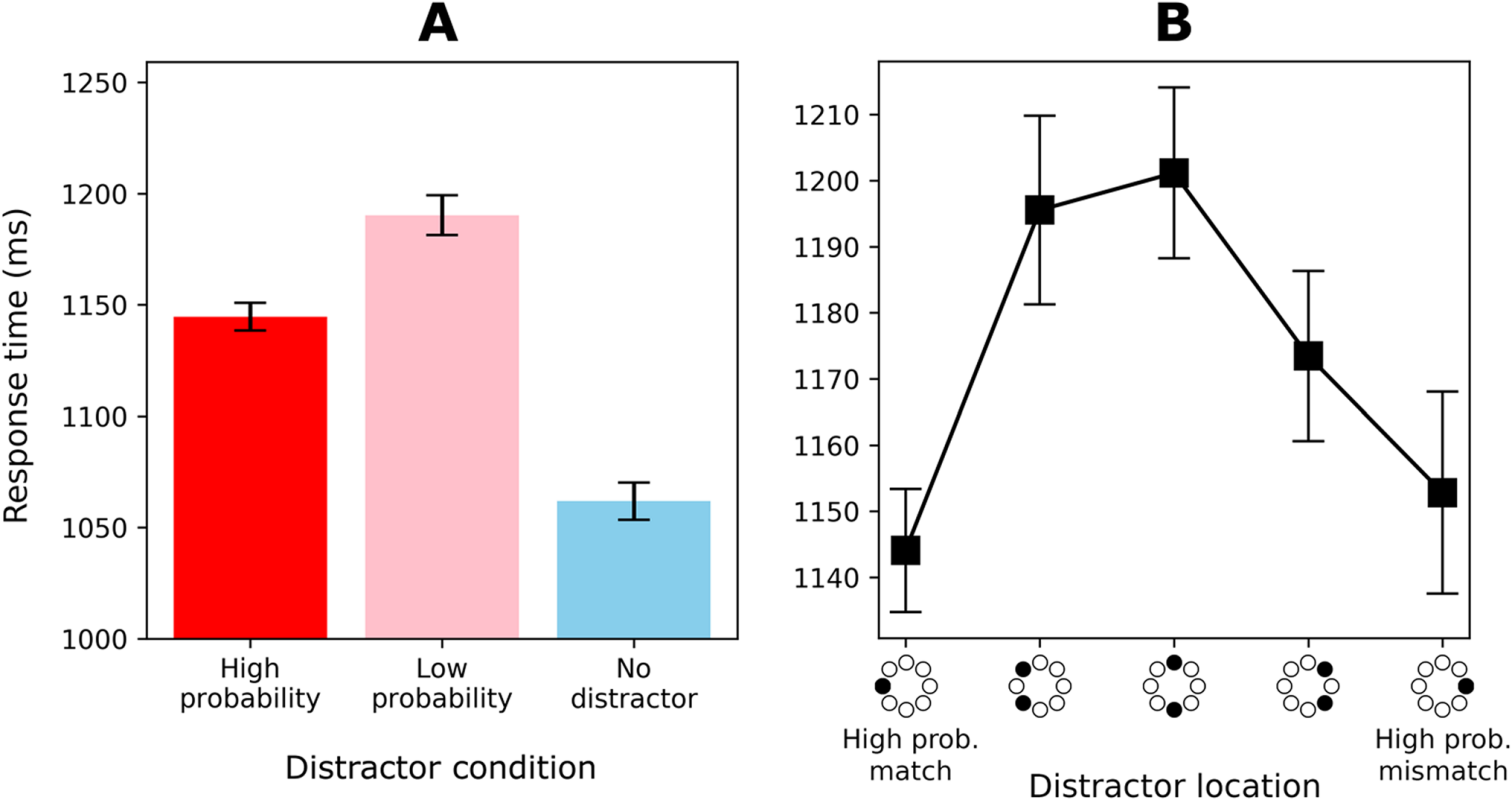
Response time (RT) results for Experiment 3. Error bars indicate 95% within-subject confidence intervals (Cousineau, 2005). (**A**) Mean RTs for high-probability, low-probability, and no-distractor conditions. Note that “high probability” refers to both high-probability locations (irrespective of context), and “low probability” refers to the six remaining locations. (**B**) Mean RTs as a function of distractor location, coded as the distance from the high-probability location of the current context (high-probability match)

Figure 7A shows mean RTs for the high-probability, low-probability, and no-distractor conditions. The factor *distractor condition* showed a main effect, *F*(1.8, 100.93) = 178, *p* < .001, partial *η*^*2*^ = 0.76. Planned comparisons showed that the distractor captured attention reliably for both the low-probability, *t*(56) = 16.2, *p* < .001, *d* = 2.15, and high-probability locations, *t*(56) = 13.73, *p* < .001, *d* = 1.82. Furthermore, they showed a reliable difference between the high- and low-probability locations, *t*(56) = 6.92, *p* < .001, *d* = 0.92, indicating that participants learned the overall regularities in the experiment.

### Is the learned distractor suppression context-dependent?

Figure 7B shows the mean RTs for distractor locations in greater detail. The comparison between high-probability match and high-probability mismatch trials was not significant, *t*(56) = 1.64, *p = .*11, *d* = 0.22, *BF* = 1.97, with the *BF* indicating that the observed data are two times more likely to have occurred under the null hypothesis of distractor suppression being context-independent. RTs for the high-probability mismatch location were faster than for the low-probability locations, *t*(56) = 7.02, *p < .*001, *d* = 0.93, indicating that the mismatch location was indeed suppressed.

### Awareness of the regularities

About half of the participants (49%) indicated that the distractor occurred more often at some locations than others. The mean awareness score across participants was 1.96 (SD = 0.52), yielding a *BF* of 10.3 in favor of no awareness. However, in contrast to Experiments 1 and 2, we found that participants who answered “yes” (aware group) on the first question had lower (i.e., more accurate) awareness scores, *t*(55) = 2.49, *p = .*016, *d* = 0.66, and the awareness scores of those participants differed significantly from chance, *t*(27) = 2.04, *p = .*025, *d* = 0.39.

**Fig. 8:**
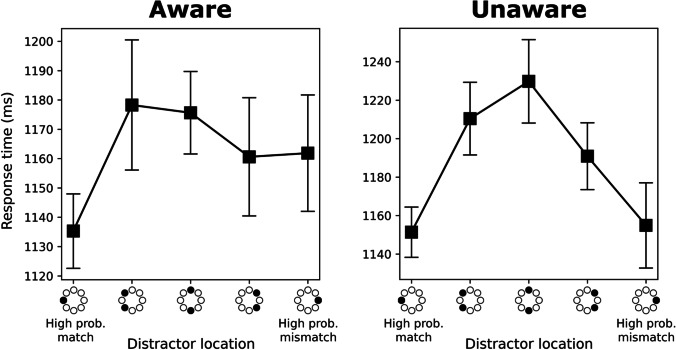
Response time (RT) results for Experiment 3, separated for the aware and unaware group. Error bars indicate 95% within-subject confidence intervals (Cousineau, 2005). Mean RTs as a function of distractor location, coded as the distance from the high-probability location of the current context (high-probability match)

We re-ran the previous RT analyses separately for the aware and the unaware group, and found that the pattern of results was largely the same, with the important difference that the comparison between the high-probability match and mismatch locations was not significant in the unaware group, *t*(28) = 0.27, *p = .*792, *BF* = 4.9, *d* = 0.05, but significant in the aware group, *t*(27) = 2.18, *p = .*038, *BF* = 0.65, *d* = 0.41. The context-dependent suppression effect in the aware group remained in place even after controlling for intertrial location priming by removing all trial-to-trial distractor location repetitions, *t*(27) = 2.37, *p = .*025, *BF* = 0.47, *d* = 0.45. Furthermore, RTs for the high-probability mismatch location were significantly faster than the low-probability locations in the unaware group, *t*(28) = 3.63, *p = .*001, *BF* = 0.03, *d* = 0.67, but not in the aware group, *t*(27) = 0.73, *p = .*472, *BF* = 3.91, *d* = 0.14 (also after controlling for intertrial priming, *t*(27) = 0.04, *p = .*967, *BF* = 4.98, *d* = 0.01), indicating that the high-probability mismatch location was not or only weakly suppressed. The separate plots for the aware and unaware groups are shown in Fig. 8. We conclude that context-dependent suppression is weak or absent when participants are not aware of the to-be-suppressed location (as in Experiments 1 and 2 and the unaware group in Experiment 3), but when participants become aware of the distractor regularities (as in the aware group of Experiment 3), they are capable of adjusting their priority map more flexibly.

## Discussion

In line with Experiments 1 and 2, Experiment 3 initially showed no context-specific suppression effects. There was clear evidence that participants learned to suppress the high-probability distractor locations, but this suppression was generalized across contexts. However, closer analysis on awareness revealed that, in contrast to Experiments 1 and 2, the group of participants that indicated that they were aware of the distractor regularity (aware group) also performed above-chance in selecting the correct distractor location per context, suggesting that there was at least some level of awareness in this group. Crucially, the aware group also showed context-dependent suppression effects: there was more suppression in the high-probability match versus mismatch condition, and the mismatch condition was not suppressed relative to the low-probability condition. This points to an advantage of awareness in context-based attentional suppression.

## Merged results

In three separate experiments, Bayesian analyses revealed that the learned distractor suppression generalized across the two contexts, and that it was learned outside the realm of awareness. One advantage of using Bayesian statistics is that results can be combined across several different experiments into a single statistic. We looked first at the combined results of Experiments 1 and 2, because in those experiments, unlike Experiment 3, processing the context was not required to perform the task. The comparison between high-probability match and high-probability mismatch trials across the first two experiments yielded a *BF* of 5.32. The same comparison across all experiments yielded a *BF* of 2.64. However, in Experiment 3 the awareness test revealed above-chance performance in the group that indicated that they noticed the distractor regularities. After excluding this “aware” group (28 participants) from the analysis across all experiments, the *BF* was 5.65, providing substantial (Jeffreys, 1998) evidence for suppression effects that are generalized across contexts, so long as participants are not aware of the distractor regularities.

## General discussion

We investigated statistical learning of distractor suppression by presenting a uniquely colored distractor item more frequently in one location than the other locations. Crucially, we investigated the context-dependency of this type of statistical learning with different context manipulations. Across three experiments we created two contexts, and each context was assigned its own high-probability distractor location. In Experiment 1 context was signified through the background color of the experimental display, in Experiment 2 context was cued at the start of a trial by a flashed ring or a tone. In Experiment 3 the context was linked to the correct response, so that participants were forced to process the context in order to be able to perform the task. It is evident that in Experiment 3 context could not have been ignored, while this may have been possible in the first two experiments. Across all experiments, participants learned to suppress the high-probability distractor locations. Notably, these learning effects occurred despite the fact that the statistical regularity was task irrelevant (i.e., it concerned the distractor instead of the target) and in participants who were likely not aware of the regularity. However, spatial suppression effects occurred independent of the context, so that the pattern of suppression reflected a de-prioritization of both high-probability locations and did not change with the context. More specifically, Bayesian analyses support the conclusion that responses were equally fast when a distractor appeared at the high-probability location matching versus mismatching with the context, so long as participants were not aware of the distractor regularities. This finding places an important limitation on implicitly learned attentional suppression, as it reveals a surprising inflexibility to adjust the suppression to a given context.

Statistical learning necessitates integration of information across multiple trials. In the present study, the only way to learn where a distractor is most likely to occur is to “track” distractor locations across a series of search displays and distill the location that occurred most often. Britton and Anderson (2020) argue that this requirement for learning can explain why learning generalizes across contexts. For context-dependent learning of distractor locations, contextual information would have to be bound to the distribution of distractor locations across a series of trials. Such a process could be a lot more demanding than simply binding information with a context on individual trials, and therefore may not take place altogether. Our findings show that when context is made more prominent (Experiments 1 and 2), and even when it is made task-relevant (Experiment 3), this binding does not occur so long as participants are unaware of the spatial regularities. In fact, Bayesian analyses provide evidence for the opposite, namely that the learned suppression occurs independent of the context. This places a severe theoretical limit on the influence of statistical learning on attentional prioritization, at least with regards to suppression. In Experiment 3, we observed some context-dependent suppression effects, but only in a subgroup of participants who had explicit knowledge of the distractor location regularities. Since modulations of capture due to learned distractor regularities are generally robust in the absence of awareness, we focus our discussion on the group of unaware participants. If suppression on the basis of implicitly (i.e., outside of awareness) learned regularities cannot be adjusted flexibly from context to context, the suppression in the current priority map must always be based on some kind of averaging from the (recent) past up until the present. In a somewhat caricatured example, one could imagine that following an experiment involving implicitly learned spatial suppression, a red traffic light is missed because it happens to occur in the suppressed location of a participant’s visual field. All in all, this would suggest a rather unintelligent learning mechanism, resulting in inefficient use of neural capacities.

In contrast to the present results, both reward- and punishment-based learning, which directly associate stimulus features with some outcome, have context-dependent effects on attentional priority (Anderson, 2015; Anderson & Kim, 2018; Grégoire et al., 2020). This is suggestive of a distinction between associative learning and statistical learning in the way they treat context. A potential explanation for this divide could be that the association between a stimulus feature and its outcome (punishment or reward) can occur on a single-trial basis, whereas associations with a high or low probability require more repetitions (and thus a kind of mental “tracking”) to be learned. Another distinction can be derived from the results of Experiment 3, relating to awareness. Specifically, the group of participants that indicated an awareness of the distractor regularities (verified through above-chance performance when indicating the high-probability locations) suppressed the distractor in a context-dependent way, whereas the “unaware” group did not. This distinction can be explained in various ways, but the most likely explanation is that participants who become aware of the most probable distractor location per context apply suppression in a more goal-directed and top-down way, allowing for greater trial-to-trial flexibility. Arguably, by the point that participants become aware of the regularity the observed effects are no longer solely in the realm of statistical learning, so that the main conclusion of this study, *statistically learned* suppression generalizes across contexts, remains unchanged. Hypothetically, a third divide could be between distractor-based and target-based learning if statistically learned target probabilities would turn out to be context-dependent. As of yet that is unknown, and all of these divides require further investigation.

Given the far-reaching consequences that a truly context-independent mechanism of statistically learned suppression would have, we must be careful in interpreting the present results. One clear limitation of the present study is that all experiments investigated context changes within the same task. Even in Experiment 3, where context was made task-relevant by binding it to the correct response, the task itself (i.e., find the unique shape and report its line orientation) remained constant throughout the experiment. In reality, by contrast, changes in context are usually accompanied by changes in the “task” one is engaged with. Britton and Anderson’s (2020) study (Experiment 3) in fact suggests that statistically learned suppression does not transfer between tasks. Furthermore, one might argue that the design of the current experiments allowed for the observed inflexibility of suppression. Simply suppressing both high-probability distractor locations irrespective of the current context was perhaps not the most refined strategy^1^ of attentional deployment, but it was also not a bad strategy. In the vast majority of trials (with the exception of trials in which a target appears on the high-probability distractor location of the other context), it was a strategy that works out very acceptably, if not optimally. Of course, attention could have been deployed even more efficiently if the spatial suppression was applied in a truly context-dependent manner, but such flexibility must also come at a computational cost. As each context was also bound by the overarching context of the experiment, it might even be seen as a useful feature of statistical learning to generalize across the two contexts. In that light, it could be valuable to investigate the learning of non-spatial features such as distractor color. This would allow for paradigms where the cost of generalization becomes much larger, and it could furthermore show whether the distractor as a whole is processed in a context-independent way. Lastly, these findings are limited to the statistical learning in the visual domain and of spatial distractor locations more specifically. Target-based statistical learning might function in ways more sensitive to context, and findings from statistical learning in the auditory domain suggest that contextual cues such as a change in voice or pitch do help listeners to track multiple sets of embedded patterns in continuous speech input (Gebhart et al., 2009; Weiss et al., 2009). Without such a cue to signify a change in context, previously learned patterns are rapidly replaced by new ones (Siegelman et al., 2018).

In sum, the present findings suggest that implicitly learned spatial suppression cannot be applied flexibly. While participants clearly learned both high probability distractor locations, they were unable to prioritize one over the other in accordance with the current context on a trial-by-trial basis, so long as they were not aware of the regularities. This finding places an important limitation on implicitly learned attentional suppression, but given the far-reaching consequences that a truly context-independent mechanism of suppression would have, some reservations are in order. The present experiments only investigated the flexibility of suppression in responses to changes in context, not the task. Furthermore, a generalized suppression strategy was still relatively sensible given our experimental setup. Lastly, our findings are limited to the implicit learning of distractor suppression, and are not in line with findings from auditory statistical learning. Future research is necessary to find out if statistical learning of spatial attention can be context-dependent, and if so, under what boundary conditions.

## Footnotes

1 Contrary to the explicit intentions that the word “strategy” suggests, the majority of the suppression observed in the present experiments was likely exercised outside the realm of awareness.
